# Magnesium Isoglycyrrhizinate Ameliorates Fibrosis and Disrupts TGF-β-Mediated SMAD Pathway in Activated Hepatic Stellate Cell Line LX2

**DOI:** 10.3389/fphar.2018.01018

**Published:** 2018-09-25

**Authors:** Jie Kai Tee, Fei Peng, Yeong Lan Tan, Bo Yu, Han Kiat Ho

**Affiliations:** ^1^NUS Graduate School for Integrative Sciences and Engineering, Centre for Life Sciences, National University of Singapore, Singapore, Singapore; ^2^Department of Pharmacy, Faculty of Science, National University of Singapore, Singapore, Singapore; ^3^Department of Pharmacy, Fudan University Shanghai Cancer Center, Shanghai, China

**Keywords:** magnesium isoglycyrrhizinate, transforming growth factor beta, Smad, hepatic stellate cells, hepatocytes, liver fibrosis

## Abstract

Liver fibrosis is a histological change often attributed to the activation of hepatic stellate cells (HSCs) and the excessive formation of scar tissues in the liver. Advanced stages of the disease frequently lead to cirrhosis. Magnesium isoglycyrrhizinate (MgIG) has been accepted as a hepatoprotective drug with the potential of alleviating inflammatory conditions and thus promote liver recovery from viral- or drug-induced injury. While MgIG has been empirically integrated into the clinics to treat some liver diseases, its anti-fibrotic effect and the associated mechanisms remain poorly characterized. Herein, we demonstrated that 1 mg/ml MgIG attenuated the production of αSMA and collagen-1 in activated HSCs using TGF-β1-induced human HSCs LX2 as the fibrotic cell model. We found that MgIG exerts an inhibitory effect on the TGF-β-SMAD signaling pathway by arresting the binding of downstream transcription factors SMAD2/3 and SMAD4. Furthermore, MgIG was shown to suppress proliferation and induce senescence of activated LX2 cells. Protein expression of p27 and enzymatic activity of senescence-associated β-galactosidase were elevated upon exposure to MgIG. In addition, we observed that exposure of activated LX2 cells to MgIG reduces TGF-β-induced apoptosis. Interestingly, a lower toxicity profile was observed when human fetal hepatocytes LO2 were exposed to the same concentration and duration of the drug, suggesting the specificity of MgIG effect toward activated HSCs. Overall, hepatoprotective concentrations of MgIG is shown to exert a direct effect on liver fibrosis through inhibiting TGF-β-signaling, in which SMAD2/3 pathway could be one of the mechanisms responsible for the fibrotic response, thereby restoring the surviving cells toward a more quiescent phenotype. This provides critical mechanistic insights to support an otherwise empirical therapy.

## Introduction

Liver fibrosis is a disease characterized by the activation of hepatic stellate cells (HSCs) and the deposition of extracellular matrix (ECM) such as collagen in response to injury (Friedman, 2004; Pellicoro et al., 2014). Morphologically, this condition is further aggravated by the loss of fenestrae in sinusoidal endothelial cells and loss of hepatocyte microvilli, thereby deteriorating the overall hepatic function (Friedman, 2000; Dechêne et al., 2010). If left untreated, the excessive accumulation of scar tissues can progress into cirrhosis and liver failure. The perisinusoidal HSCs which are responsible for the deposition of ECM have been the primary focus in treating liver fibrosis (Higashi et al., 2017). Since the activation of HSCs remains as the central event of hepatic fibrogenesis, recent therapeutic approaches to revert HSCs back to its quiescent state could potentially promote fibrosis regression or even reversal of fibrosis (Friedman and Bansal, 2006; Friedman, 2012). Although novel strategies with the potential to alleviate the fibrotic condition such as targeting kinase receptors including transforming growth factor beta receptor (TGFβR) and fibroblast growth factor receptor (FGFR), promotion of ECM degradation and cell-based therapies have begun to emerge over the past few years, inadequate scientific insights and implementation challenges persist (Lee et al., 2015; Wang et al., 2016; Lim et al., 2017). To date, liver transplantation remains the only treatment option for patients with advanced liver fibrosis (Durand and Francoz, 2017).

Nutraceuticals present a new therapeutic approach to treat liver diseases (Del Ben et al., 2017). Magnesium isoglycyrrhizinate (MgIG) is one of such compounds whose anti-fibrotic potential was first presumed based on its hepatoprotective properties. Previous studies have shown that MgIG protects hepatic cells from reperfusion-induced injury (Huang et al., 2014) and hypoxia-reoxygenation injury (Zheng et al., 2015). In addition, studies on either non-alcoholic or alcoholic liver diseases (NALD or ALD) have indicated the potential of MgIG in protecting hepatic cells against fatty acid-induced lipotoxicity (Cheng et al., 2009) and steatosis (Lu et al., 2017). Recently, MgIG has also been shown to exert its hepatoprotective effect against compounds such as cyclophosphamide (Jiang et al., 2017) and doxorubicin (Wu et al., 2018) by increasing the activity of anti-oxidant enzymes and reducing the inflammatory response. Mechanistically, studies have shown that the anti-inflammatory activity of MgIG is attributed to its potential to inhibit inflammatory-associated pathways such as STAT3 (Tang et al., 2015), phospholipase A_2_/arachidonic (Xie et al., 2015) and NF-κB pathways (Jiang et al., 2017; Zhao et al., 2017). Taken together, these studies have illustrated the hepatoprotective effects of MgIG in various liver injuries.

Conversely, there is a lack of studies with direct focus on the effect of MgIG on the microenvironment of liver fibrosis, particularly on the HSCs. Recently, Bian et al. (2017) have reported that MgIG induced cell cycle arrest and promoted apoptosis in HSC-T6 cells. In addition, they have shown that MgIG could alleviate liver fibrotic injury via the activation of endoplasmic reticulum stress signaling pathway. However, there were no mechanistic studies of MgIG effect in the transforming growth factor beta (TGF-β)-activated HSCs. TGF-β plays a pivotal role in regulating liver diseases, driving key signaling processes that exacerbates liver injury, fibrosis and eventually cirrhosis (Lewindon et al., 2002; Dooley and ten Dijke, 2012). We postulated that any significant therapeutic effect of MgIG must negotiate the TGF-β-SMAD signaling pathway, which mediate the actual biochemical process of fibrogenesis. These changes would then translate into modulation of the expression and deposition of proteins such as alpha-smooth muscle actin (αSMA) and collagen-1 in activated HSCs. In addition, the suppression of TGF-β signaling could potentially revert HSCs back to its quiescent state through the induction of senescence as a safer biochemical route toward inhibiting fibrogenesis and promoting hepatic recovery.

## Materials and Methods

### Chemicals and Reagents

Human HSC LX2 was received as a kind gift from Prof. Scott Friedman. Human fetal hepatocytes LO2 was received as a kind gift from A/Prof. Victor Yu. MgIG was obtained from Jiangsu Chia-Tai Tianqing Pharmaceutical Co., Ltd. (Nanjing, China). Compound structure and detailed information is supplied in **Supplementary Figure S1**. PCR primers were purchased from Integrated DNA Technologies (Coralville, IA, United States). The following antibodies were used: Anti-collagen-1 was purchased from Abcam (Cambridge, United Kingdom), anti-αSMA from Agilent Dako (Santa Clara, CA, United States); anti-GAPDH, anti-phospho-ERK, anti-ERK, anti-phospho-Akt, anti-Akt, anti-phospho-JNK, anti-JNK, anti-SMAD2/3, anti-SMAD4, secondary anti-mouse and anti-rabbit were purchased from Cell Signaling Technology (Danvers, MA, United States); anti-phospho-p38, anti-p38 and anti-p27 were purchased from Santa Cruz Biotechnology (Dallas, TX, United States). 3-(4,5-Dimethylthiazol-2-yl)-2,5-Diphenyltetrazolium Bromide (MTT) was purchased from Duchefa Biochemie (Haarlem, Netherlands) and propidium iodide (PI) was purchased from Sigma-Aldrich (St. Louis, MO, United States). 96-well cellular senescence assay kit was purchased from Cell Biolabs (San Diego, CA, United States).

### Cell Line and Culture Conditions

LX2 cells were recovered in Dulbecco’s Modified Eagle’s medium (DMEM) (Sigma-Aldrich, St. Louis, MO, United States) supplemented with 10% fetal bovine serum (FBS) and maintained in DMEM supplemented with 1% FBS. LO2 cells were maintained in DMEM supplemented with 10% FBS. Cells were incubated at 37°C in a humidified atmosphere supplied with 5% CO_2_. For TGF-β1 (Merck Millipore, Burlington, MA, United States) stimulations, LX2 cells were activated with 2 ng/ml TGF-β1 in DMEM supplemented with 1% FBS for 24, 48, and 72 h. MgIG stock solution was prepared by dissolving in culture media containing 1% FBS. For MgIG treatment, cells were pre-treated with 1 mg/ml MgIG in the respective medium for 30 min before the addition of TGF-β1.

### Quantitative RT-PCR

Total RNA was extracted using RNeasy Mini Kit (Qiagen, Hilden, Germany) according to the manufacturer’s instructions. 500 ng of RNA was then transcribed into complementary DNA (cDNA) using qScript^TM^ cDNA Supermix (Quantabio, Beverly, MA, United States). Subsequently, the cDNA obtained was diluted five times with RNase-free water and quantified for gene expression levels using QuantiFast SYBR^®^ Green Supermix (Qiagen, Hilden, Germany). The following primers were used: αSMA (forward: 5′-CCGGGAGAAAATGACTCAAA-3′, reverse: 5′-GCAAGGCATAGCCCTCATAG-3′), collagen-1 (forward: 5′-CCTGGATGCCATCAAAG TCT-3′, reverse: 5′-CGC-CATACTCGAACTGGAAT-3′) and glyceraldehyde-3-phosphate dehydrogenase (GAPDH) (forward: 5′-ACTTTGGTATCGTGGAAGGACT-3′, reverse: 5′-GTAGAGGCAGGG-ATGATGTTCT-3′) which was used as the housekeeping gene for normalization.

### Western Blotting and Immunoprecipitation

Cells lysates were extracted with radioimmunoprecipitation assay (RIPA) buffer containing 0.1% sodium dodecyl sulfate (SDS), 1% NP-40 and 0.5% sodium deoxycholate in phosphate-buffered saline (PBS). The samples were then mixed with loading dye before adding into a 10% SDS-PAGE polyacrylamide gel (Bio-Rad Laboratories, Hercules, CA, United States). Gel electrophoresis was run at 130V for 1.5 h. Subsequently, a wet transfer “sandwich” method was used to transfer the proteins from the gel onto PVDF membrane (Thermo Fisher Scientific, Waltham, MA, United States) at 4°C with 100V for 2 h. The membrane was washed with Tris-buffered saline (1st Base, Singapore) containing 0.1% Tween (TBST), blocked with 5% bovine serum albumin (BSA) and incubated with primary antibody (1:1,000) containing 2% BSA at 4°C overnight. The membrane was washed thrice with TBST, incubated with secondary antibody (1:10,000) at room temperature for 1 h and exposed with Western Lightning Plus-ECL reagent (Perkin Elmer, Waltham, MA, United States) using G:Box Gel imaging system (Syngene, Bangalore, India). Original images of Western blot analyses are supplied in **Supplementary Figure S6**.

For immunoprecipitation of SMAD proteins, primary antibody (1:100) was added into the cell lysates and incubated at 4°C overnight. Subsequently, 30 μl of protein G Sepharose Fast Flow Beads (GE Healthcare, Chicago, IL, United States) were added and the samples were further incubated at 4°C for 4 h. The beads were centrifuged at 3,000 rpm for 1 min, washed with cold RIPA buffer for three times and boiled with RIPA buffer containing SDS and dithiothreitol (DTT) at 95°C for 10 min. Finally, the beads were further centrifuged to collect the supernatant for Western blot analyses.

### Confocal Imaging

Five thousand cells per well were seeded onto 8-well chamber slide (Thermo Fisher Scientific, Waltham, MA, United States) overnight before treatment with MgIG and TGF-β for 30 min. Subsequently, 4% paraformaldehyde was used to fix the cells for 15 min, 0.2% Triton X-100 to permeabilize the cells for 15 min, 2% BSA in cold PBS to block the cells for 1 h, and lastly incubated with SMAD2/3 primary antibody (1:200) diluted in PBS containing 0.1% Triton X-100 and 0.2% BSA at 4°C overnight. The fixed cells were washed with PBS and subsequently incubated with chicken anti-mouse/rabbit antibody (1:400) (Thermo Fisher Scientific, Waltham, MA, United States) together with phalloidin (Biotium, Fremont, CA, United States) and Hoechst 33342 dye (Sigma-Aldrich, St. Louis, MO, United States) at room temperature for 1 h. Cells were washed again with PBS and mounted with Fluoromount^TM^ aqueous mounting medium (Sigma-Aldrich, St. Louis, MO, United States). Fluorescence images were taken with Olympus Fluoview FV1000 confocal microscope (Olympus, Tokyo, Japan). JACoP ImageJ (Bolte and Cordelieres, 2006) was used to calculate the degree of colocalization.

### MTT Assay

Five thousand cells per well were seeded in a 96-well plate and left overnight before treatment with MgIG and TGF-β for the stated duration. At each timepoint, the treatment media was removed and 0.5 mg/ml MTT solution was added to the cells for 3 h at 37°C. Thereafter, the MTT solution was removed and 200 μl of dimethyl sulfoxide (DMSO) was added to dissolve the purple formazan dye. The plate was shaken for 5 min at room temperature and absorbance was measured at 570 nm wavelength using Hidex sense microplate reader (Hidex, Turku, Finland). The average absorbance from three biological replicates was then plotted against the treatment time at 24-h intervals.

### Propidium Iodide Staining and Cell Cycle Analysis

Cells were seeded at a density of 20,000 cells per cm^2^ onto a 6-well plate overnight before the addition of TGF-β and MgIG. After 24, 48, and 72 h treatment, both the adhered live cells and floating dead cells were harvested, washed with cold PBS and mixed with 300 μl of PBS containing 10 μg/ml PI stain. The percentage of dead cells was then determined with Beckman Coulter CyAn ADP flow cytometer (Beckman Coulter, Brea, CA, United States). Three biological replicates were performed and the average percentage of dead cells was plotted against treatment time. For cell cycle analysis, the cells were further permeabilized with 70% ethanol at 4°C for 1 h. Thereafter, the cells were sorted up to 20,000 events with FC 500 flow cytometer (Beckman Coulter, Brea, CA, United States) and assessed based on the DNA quantity. Nuclei number was then plotted against the measured DNA fluorescence. Non-treated cells were used as the negative control to determine the percentage of cells in different cell cycle phases.

### β-Galactosidase Activity Assay

β-Galactosidase activity assay was performed according to the manufacturer’s instructions (Cell Biolabs, San Diego, CA, United States). Briefly, 10,000 cells were seeded into 96-well plate overnight and the respective treatment media was added. The cells were then lysed with the provided cell lysis buffer and the extracted protein lysates were further incubated with senescence associated (SA) β-galactosidase substrate at 37°C for 1 h. Subsequently, the samples were transferred onto a 96-well black plate and fluorescence signal was measured at excitation/emission wavelength of 390/460 nm using Hidex sense microplate reader (Hidex, Turku, Finland). The average fluorescence was then plotted against different treatment conditions at 24-h intervals.

### Apoptosis Detection

Ac-DEVD-AMC Caspase-3 Fluorogenic Substrate (BD Pharmingen, United States) was used to assess caspase-3 activity in MgIG-treated cells. Cells were seeded at a density of twenty thousand cells per cm^2^ onto a 6-well plate overnight before treatment with TGF-β and three different concentrations of MgIG (0.5 mg/ml, 1.0 mg/ml, and 5.0 mg/ml) for 24, 48, and 72 h. After each timepoint, the cells were harvested and lysed in RIPA buffer for 15 min. 100 μg of protein lysate was added to 300 μl of protease assay buffer (2 mM DTT, 10% glycerol, 20 mM HEPES, 20 μM Ac-DEVD-AMC substrate) and the samples were further incubated at 37°C in the dark for 1 h. 100 μl of samples were then added into 96-well black plate and the fluorescence intensity was quantified based on excitation/emission wavelength of 390/460 nm using Hidex sense microplate reader (Hidex, Turku, Finland). The average fluorescence was then plotted against different treatment conditions at 24-h intervals.

### Statistical Analysis

Statistical significance was calculated based on a one-way analysis of variance (ANOVA) with Tukey HSD test, unless otherwise stated. Calculated *p*-value < 0.05 is denoted as statistically significant. Data represents mean ± SE (*n* = 3).

## Results

### MgIG Reduced Fibrogenesis in Activated LX2 Cells

**FIGURE 1:**
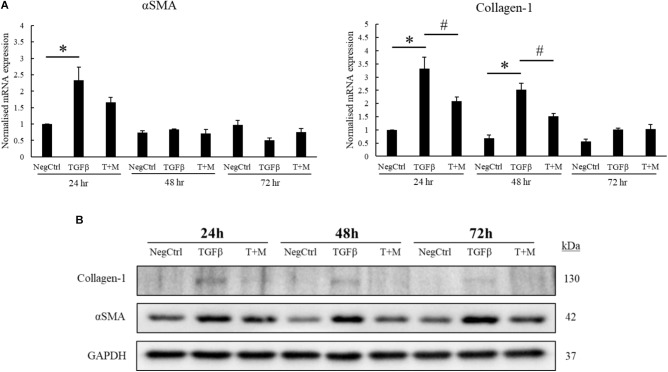
MgIG reduced expression of fibrotic markers in TGF-β-activated hepatic stellate cells LX2. **(A)** 1 mg/ml MgIG inhibited TGF-β-induced mRNA expression of both αSMA and collagen-1 at 24 h treatment (T+M), although the decrease in αSMA expression was not statistically significant. The increase in collagen-1 mRNA expression at 48 h treatment was also found to be significantly inhibited by MgIG. Data represents means ± SE of three biological replicates, one-way ANOVA with Tukey HSD test, *p*-value < 0.05, ^∗^significance against non-treated control (NegCtrl), ^#^significance against TGF-β treatment. **(B)** Western blot analyses detected a reduction in protein levels of both αSMA and collagen-1 after treatment with 1 mg/ml MgIG up to 72 h.

The activation of HSCs by TGF-β contributes significantly to the progression of liver fibrosis through the upregulation of αSMA and excessive production of collagen-1 (Lewindon et al., 2002; Dooley et al., 2003). In accordance with this concept, we optimized our fibrotic cell model by treating LX2 cells with increasing concentrations of TGF-β for 24 h. As shown in the mRNA analyses, the expression of αSMA and collagen-1 plateaued at 2 ng/ml TGF-β but did not further increase at higher concentration (5 ng/ml) of the growth factor (**Supplementary Figure S2A**). Although the protein expression profile of both αSMA and collagen-1 did not correlate completely with their respective mRNA levels, TGF-β was still found to increase both fibrotic markers at concentrations up to 5 ng/ml (**Supplementary Figure S2B**). Based on the mRNA analyses, we fixed the concentration of TGF-β used for subsequent experiments to be 2 ng/ml. We tested our hypothesis on whether MgIG could perturb the expression of TGF-β-induced fibrotic markers by treating the cells with either TGF-β alone or TGF-β concurrently with MgIG for up to 72 h. Importantly, cells treated with co-treatment showed significant reduction in both αSMA and collagen-1 mRNA levels at 24 h compared to TGF-β treatment alone (**Figure 1A**). Furthermore, the increase in mRNA levels of collagen-1 at 48 h was also significantly reduced by MgIG. In addition, our Western blot analyses showed that MgIG reduced the protein expression of both fibrotic markers up to 72 h (**Figure 1B**). Notably, the decrease in protein levels of αSMA in the presence of MgIG was the most significant at 48 h timepoint. Nevertheless, the addition of MgIG was observed to suppress both the mRNA and protein levels of the fibrotic markers, suggesting the potential inhibitory effect of MgIG in TGF-β-induced fibrosis.

### MgIG Disrupts TGF-β-SMAD Signaling Pathway

**FIGURE 2:**
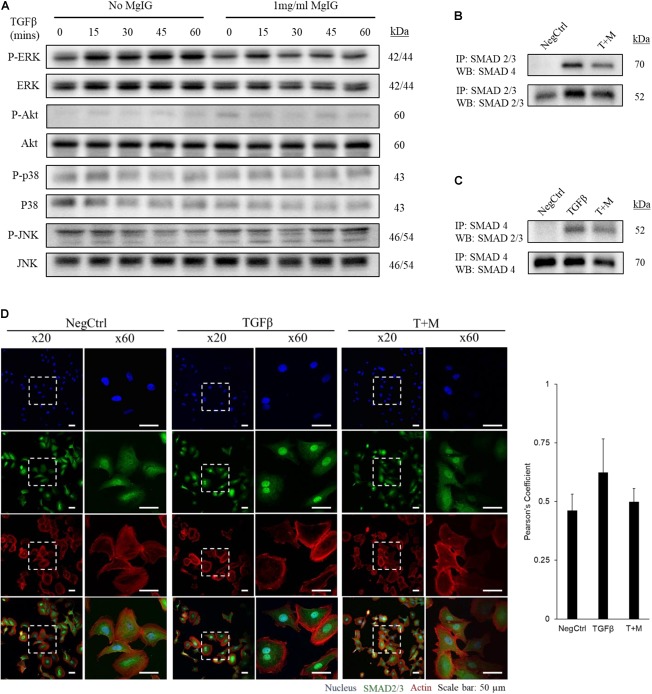
MgIG inhibited TGF-β-induced SMAD2/3 nuclear localization through blocking of ERK pathway. **(A)** LX2 cells treated with MgIG exhibited a reduction in phosphorylated ERK up to 60 min. There were no observable effects in other major downstream pathways of TGF-β signaling. After 30 min pre-treatment of cells with 1 mg/ml MgIG, TGF-β was added into the culture media and the cells were harvested at 15, 30, 45, and 60 min for protein analysis. **(B**) Immunoprecipitation was performed to pull down SMAD2/3. Western blot analyses revealed increase in SMAD4 binding to SMAD2/3 after 30 min treatment with TGF-β. MgIG was found to reduce SMAD4 binding to SMAD2/3. **(C)** SMAD4 was pulled down to reveal binding of SMAD2/3 after TGF-β treatment for 30 min. MgIG was also found to reduce the binding of the SMAD proteins. **(D)** Immunofluorescence staining of TGF-β-activated LX2 cells after 30 min treatment showed an increase in nuclear localization of SMAD2/3 as viewed under a confocal microscope at ×20 and ×60 magnification. 1 mg/ml MgIG was found to reduce SMAD2/3 localization to the nucleus. SMAD2/3 antibody was tagged with Alexa488 (green), actin stained with CF568 phalloidin (red) and nucleus stained with Hoechst dye (blue). The degree of colocalization between SMAD2/3 and the nucleus was analyzed with ImageJ using JACoP and Pearson’s coefficient was calculated. Data represents means ± SD of three image fields.

We observed that MgIG reduced the mRNA expression of αSMA and collagen-1 (**Figure 1A**), suggesting that its inhibitory effect may lie upstream of such gene transcriptions. To elucidate this mechanism, we first explored various major pathways which may mediate the signaling of TGF-β (Zhang, 2009). Among the key pathways investigated, only phosphorylated ERK was found to be elevated with the addition of TGF-β within the stimulated time points of 15 min-intervals (**Figure 2A**). Interestingly, MgIG was able to inhibit the phosphorylation of ERK up to 60 min. ERK pathway plays a pivotal role in mediating TGF-β-SMAD signaling, thereby activating transcriptional factors SMAD2 and SMAD3, which then localized to the nucleus to regulate gene expression (Hough et al., 2012; Principe et al., 2017). Therefore, we further investigated the effect of TGF-β on the canonical SMAD2/3 and SMAD4 protein binding, a gateway process required for the nuclear translocation of SMAD proteins to regulate transcription activity (Gaarenstroom and Hill, 2014). The binding of SMAD2/3 to SMAD4 was observed when cells were treated with TGF-β for 30 min (**Figures 2B,C**). In addition, MgIG was found to partially reduced the binding of the SMAD proteins. To further confirm these findings, we performed immunofluorescence staining to visualize the cellular localization of SMAD2/3 after 30 min treatment with either TGF-β alone or both TGF-β and MgIG (**Figure 2D**). We observed that TGF-β-induced localization of SMAD2/3 to the nucleus at 30 min. Compared to the TGF-β treatment alone, cells treated with both TGF-β and MgIG showed a reduction in the nuclear localization of SMAD2/3. This difference was not observed when the treatment duration was reduced to 15 min (**Supplementary Figure S3**). In line with the immunoprecipitation data of SMAD2/3 and SMAD4 binding, these results suggested that MgIG could have blocked the TGF-β signaling pathway through the inhibition of upstream ERK and SMAD activation as an early event, thereby reducing the transcriptional activity of SMAD proteins.

### MgIG Reduced Proliferation of Activated LX2 Cells

**FIGURE 3:**
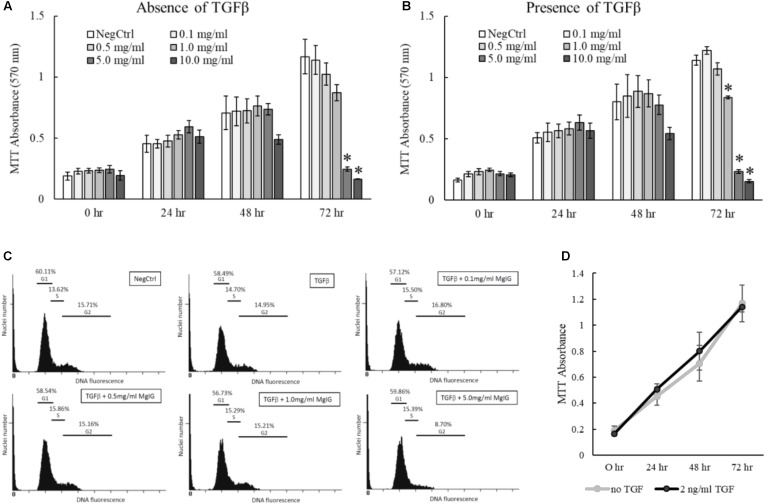
Anti-proliferative effects of MgIG on TGF-β-activated LX2 cells. **(A)** MgIG reduced proliferation of LX2 cells in a dose-dependent manner up to 72 h treatment. Data represents means ± SE of three biological replicates. ^∗^Two-tailed *t*-test with *p*-value < 0.05 compared to negative control (NegCtrl) of respective time point **(B)** MgIG exhibited similar anti-proliferative profile in TGF-β-activated LX2 cells. Data represents means ± SE of three biological replicates. ^∗^Two-tailed *t*-test with *p*-value < 0.05 compared to TGF-β-treated control (NegCtrl) of respective time point **(C)** Cell cycle analysis of LX2 cells treated with 5 mg/ml MgIG for 24 h showed reduction of cells at the G2 phase. **(D)** Treatment with TGF-β did not show any significant change in proliferation of LX2 cells up to 72 h. Data represents means ± SE of three biological replicates.

The proliferation of activated HSCs exacerbates the progression of liver fibrosis (Li et al., 2017). By inhibiting the proliferation of HSCs, it is anticipated that further deposition of scar tissue can be arrested to allow hepatic recovery to take place. Accordingly, we tested different concentrations of MgIG on LX2 proliferation and found that higher concentrations of MgIG (5 and 10 mg/ml) could significantly reduce the number of cells in both quiescent and activated LX2 populations (**Figures 3A,B**). Since a higher concentration of MgIG (5 mg/ml) was able to reduce the proliferation of activated LX2 cells, we asked whether the anti-proliferative effect could be attributed to changes in the cell cycle of the proliferating cells. To test our hypothesis, we further analyzed the different cell cycle phases by assessing the DNA content in the cells. Conceptually, proliferative cells at the G2 growth phase would exhibit higher DNA content (Nusse et al., 1990). In accordance to our MTT results, we found that high concentration of MgIG (5 mg/ml) reduced the percentage of cells in the G2 phase from 14.95% in TGF-β treatment alone to 8.7% after 24 h (**Figure 3C**). Notably, as the cells were cultured in low serum conditions of DMEM with 1% FBS (Xu et al., 2005), we would generally expect to see low percentages of cells in the G2 phase (**Figure 3C**).

Transforming growth factor beta is a multifunctional growth factor that supports proliferation, differentiation, ECM production and adhesion of LX2 cells (Khimji et al., 2008). However, we found that 2 ng/ml TGF-β did not increase the percentage of cells in the G2 phase (**Figure 3C**) or promote the proliferation of LX2 cells up to 72 h (**Figure 3D**). We postulated that TGF-β signaling could have diverged to other pathways that induce other phenotypic changes such as the induction of fibrotic markers (**Figures 1A,B**) and the nuclear localization of SMAD proteins (**Figures 2B–D**). In addition, the low serum conditions used to maintain the quiescent state of LX2 cells could also contribute to the slow growth phenotype of the cells. Nevertheless, these data ascertained that MgIG exhibits anti-proliferative effects in LX2 cells.

### MgIG Induced Senescence in Activated LX2 Cells

**FIGURE 4:**
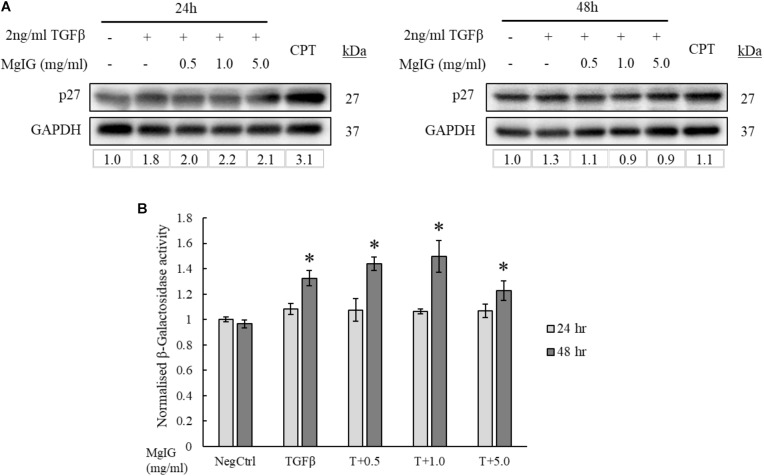
MgIG increased p27 expression and β-galactosidase activity in TGF-β-activated LX2 cells. **(A)** Western blot and densitometric analysis (against GAPDH loading control) of the proteins showed that MgIG induced expression of p27 senescence marker particularly at 24 h treatment. Cells treated with 5 μM camptothecin for 24 and 48 h, respectively, were used as positive controls. **(B)** TGF-β-activated LX2 cells treated with three different concentrations of MgIG for 48 h exhibited an increase in β-galactosidase activity. Data represents means ± SE, *n* = 3, one-way ANOVA with Tukey HSD test, *p*-value < 0.05, ^∗^significance against non-treated control (NegCtrl) at 48 h.

Cellular senescence is defined as the lack of proliferative potential in cells with irreversible cell cycle status (Kuilman et al., 2010; van Deursen, 2014; Fridlyanskaya et al., 2015). Since MgIG was previously known for its anti-proliferative effect on HSCs (Bian et al., 2017), we queried if the cells could have acquired replicative senescence. Consistent with Bian et al. (2017), p27 protein expression was found to be increased with MgIG treatment (**Figure 4A**). However, the increase in p27 was only observable at shorter duration of 24 h, suggesting that senescence is an early process acquired by the cells upon exposure to MgIG. The presence of β-galactosidase enzymatic activity detectable only at pH 6.0 is the most distinctive indicator of cellular senescence (Althubiti et al., 2014). It is a consequence of lysosomal enlargement in senescence cells and the enzyme responsible is often coined as senescence-associated β-galactosidase (SA-β-Gal). Therefore, to further validate our Western blot data, we further assessed the SA-β-Gal activity in MgIG treated cells at these two timepoints of 24 and 48 h. We detected an increase in SA-β-Gal activity only after 48 h (**Figure 4B**). We reasoned that SA-β-Gal activity could have been a late onset of senescence at least in part to the delayed increase in expression of the lysosomal β-galactosidase protein (Lee et al., 2006), whereas p27 represents an upstream signaling mechanism which is triggered earlier upon MgIG exposure (Hnit et al., 2015). Regardless of the treatment duration, MgIG may further commit LX2 cells to senescence in the presence of TGF-β activation.

### MgIG Induced Cell Death in TGF-β-Activated LX2 Cells Independent of Caspase Activity

**FIGURE 5:**
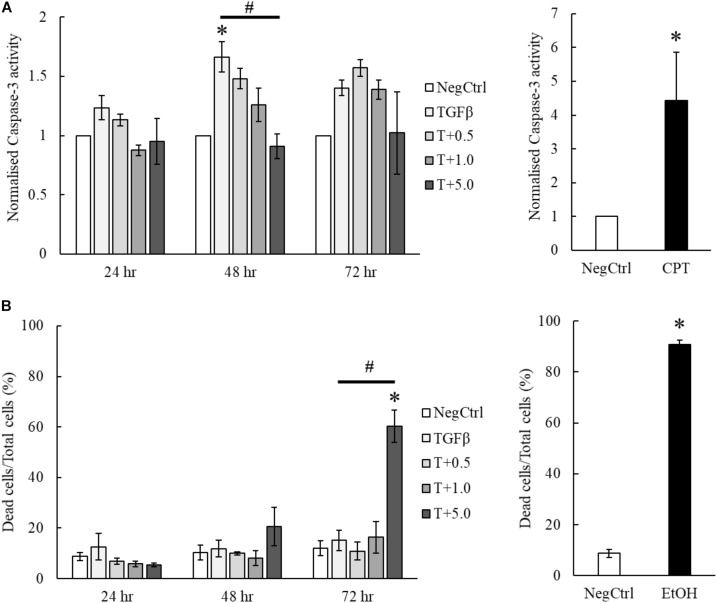
MgIG partially suppressed apoptotic cell death of TGF-β-activated LX2 cells. **(A)** MgIG was observed to reduce caspase-3 activity in TGF-β-activated LX2 cells significantly at 48 h treatment. TGF-β was shown to increase caspase-3 activity for all three timepoints. 5 μM camptothecin was used as a positive control. Data represents means ± SE of three biological replicates, one-way ANOVA with Tukey HSD test, *p*-value < 0.05, ^∗^significance against non-treated control (NegCtrl) at the respective timepoint, ^#^significance against TGF-β treatment. **(B)** PI staining on TGF-β-activated LX2 cells treated with 5 mg/ml MgIG showed a significant increase in cell death at 72 h. Cells treated with 100% cold ethanol for 5 min was used as the positive control (EtOH). Data represents means ± SE of three biological replicates, one-way ANOVA with Tukey HSD test, *p*-value < 0.05, ^∗^significance against non-treated control (NegCtrl) at the respective timepoint.

Our results indicated that the treatment of MgIG reduced the proliferation of TGF-β-activated LX2 cells (**Figure 3B**). Furthermore, we observed that the cells have committed to senescence in the presence of MgIG. This led us to hypothesize that only cells which survived through the MgIG treatment have reverted to a senescence-like state. To test this hypothesis, we first harvested adherent cells treated with MgIG and analyzed the propensity of cells to undergo cellular death. Cleaved Ac-DEVD-AMC fluorogenic substrate was used to assess the caspase-3 activity in the cells. Interestingly, we found that TGFβ induced caspase-3 activity in LX2 cells at all three time points (**Figure 5A**). This increase was reduced by the addition of MgIG in a dose-dependent manner, an observation that was clearly detectable particularly at 48 h treatment time. Although we anticipated that the cells might undergo apoptosis during longer treatment duration (72 h), cells treated with the highest concentration (5 mg/ml) did not exhibit a significantly higher caspase-3 activity. Hence, we postulated that some of the cells might have undergo necrosis and detached from the surface, or that the cells may have undergone a caspase-independent cell death. To validate this point, we harvested both the floating dead cells and adhered live cells to test for propidium iodide staining. Interestingly, we observed significant cellular death at 72 h when the cells were treated with 5 mg/ml MgIG compared to the non-treated (NegCtrl) and TGF-β alone treated cells (**Figure 5B**). When we visualized the cells using light microscopy, we also observed shrinkage of the LX2 morphology, particularly in the presence of 5 mg/ml MgIG at 72 h (**Supplementary Figure S4**). Therefore, these data further support the idea that MgIG could have reduced the apoptotic activity in some of the cells while promoting others to undergo cellular death independent of caspase activity.

### MgIG Exhibits Lower Toxicity to Hepatocytes LO2

**FIGURE 6:**
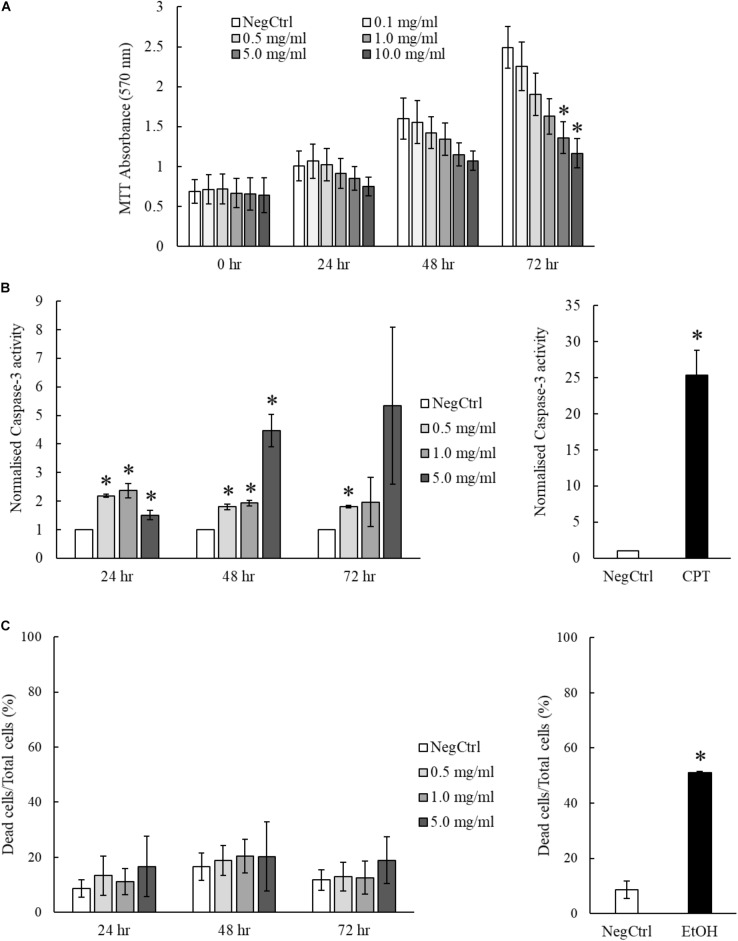
MgIG reduced proliferation of LO2 cells but did not result in significant cell death. **(A)** MgIG reduced proliferation of LO2 cells in a dose dependent manner at 72 h. Data represents means ± SE of three biological replicates. ^∗^Two-tailed *t*-test with *p*-value < 0.05 compared to negative control (NegCtrl) of respective time point. **(B)** LO2 cells treated with MgIG exhibited higher caspase-3 activity, particularly at high concentration of MgIG (5 mg/ml). 5 μM camptothecin was used as a positive control. Two-tailed *t*-test with *p*-value < 0.05, ^∗^significance against non-treated (NegCtrl) of respective time point. **(C)** PI staining revealed that MgIG did not induce significant cell death up to 72 h treatment. Cells treated with 100% cold ethanol for 5 min was used as the positive control (EtOH).

The hepatoprotective effects of MgIG have been well reported in previous studies which investigated ischemia/reperfusion-induced (Huang et al., 2014) or cyclophosphamide-induced hepatic injuries (Jiang et al., 2017). However, we showed that MgIG could exert inhibitory effects on the growth of LX2 cells, promoting their senescence or committing them to cell death. To ascertain the impact of concurrent exposure of hepatocytes to MgIG during treatment of HSC *in vivo*, we further characterize the effects of MgIG on human fetal hepatocytes LO2. We first tested the same concentrations of MgIG used in our activated LX2 cell model onto the LO2 cells. Noticeably, we observed a significant reduction in proliferation of LO2 cells up to 72 h, particularly with higher concentrations (5 and 10 mg/ml) of MgIG (**Figure 6A**). However, this decrease was not as substantial as compared to the viability results obtained from LX2 cells (**Figures 3A,B**). We further assessed the capacity of MgIG to induce apoptosis in the LO2 cells. Unexpectedly, MgIG induced caspase-3 activity especially when higher doses were used (**Figure 6B**). However, we further viewed the morphology of the cells and observed no apparent signs of apoptosis (**Supplementary Figure S5**). Hence, we performed PI staining to assess whether the hepatocytes were indeed undergoing cellular death. Cells treated with concentrations up to 5 mg/ml MgIG were shown to be viable with less than 20% cell death (**Figure 6C**), suggesting that the increase in caspase-3 activity in the presence of MgIG did not translate to overt cellular death. Moreover, comparisons with the PI staining profile of LX2 cells (**Figure 5B**) showed that the cytotoxic effect of MgIG displayed in LX2 cells is limited in LO2 cells. Hence, these results further illustrated the lower toxicity of MgIG in hepatocytes as opposed to the effects observed in activated HSCs.

## Discussion

In this study, we showed that MgIG reduced the expression of TGF-β-induced fibrotic markers, (**Figures 1A,B**) and disrupted the SMAD signaling pathway in activated human HSCs LX2 (**Figure 2**). In addition, we performed cellular assays which ascertained the anti-proliferative (**Figure 3B**) and pro-senescence effects (**Figures 4A,B**) of MgIG, a finding that concurs with a recent study of MgIG on rat HSCs HSC-T6 (Bian et al., 2017). This effect is cell-specific whereby treating human fetal hepatocytes LO2 with MgIG elicited lower percentages of cellular death compared to the activated LX2 cells (**Figure 6C**). Therefore, it could translate into a therapeutic advantage with a reduced off-target cytotoxicity on the adjacent liver parenchymal cells. That said, high concentrations of MgIG (5 and 10 mg/ml) may result in reduced cell proliferation and significant cell death, which could potentially delay liver regeneration and thus hepatic recovery (Michalopoulos, 2017). Hence, while MgIG could potentially exert its anti-fibrotic effects on the targeted HSCs, balancing this against the cytotoxic effect on the liver microenvironment, such as the neighboring hepatocytes, is of importance as well to further promote the safe use of MgIG in the treatment of liver fibrosis.

Hepatic stellate cells reside within the space of Disse between the liver sinusoidal endothelial cells and parenchymal cells of the hepatic lobule (Shang et al., 2018). Although the exact role of quiescent HSCs remains unclear, they are generally known to function as vitamin-A storing cells which maintain the normal basement membrane matrix and mediate the hepatic innate immune system (Weiskirchen and Tacke, 2014; Wilson et al., 2014). Therefore, their physiological role should be maintained while considering HSC as a target for anti-fibrotic therapy. Previous studies using concentrations up to 10 mg/ml have shown its anti-proliferative and apoptotic effect in HSCs via the induction of endoplasmic reticulum stress (Bian et al., 2017). On the other hand, the same group has saliently pointed out that the same concentrations of MgIG could also protect hepatocytes against ethanol-induced steatosis and apoptosis via the blockage of hedgehog pathway (Lu et al., 2017). However, there are currently no studies to support the idea that MgIG has the potential for specific cell targeting. Since liver fibrosis is a dynamic disease involving an array of phenotypic changes in different cells, one may speculate that high doses of MgIG could result in indiscriminate apoptosis of various cell types present, which could potentially delay liver recovery even if fibrosis is suppressed. In our study, we chose to use a low concentration of MgIG (1 mg/ml) to suppress proliferation of activated HSCs (**Figure 3B**) to allow the remaining HSCs to revert back to its quiescent state. This is further supported by our mRNA and Western blot analysis (**Figures 1A,B**) which showed the reduction of TGF-β-induced fibrotic markers by MgIG. Longer term studies in a different cell or animal model will be performed to validate this notion. Nevertheless, our study emphasized the importance of MgIG in delaying the progression of liver fibrosis through the regulation of activated HSCs.

**FIGURE 7:**
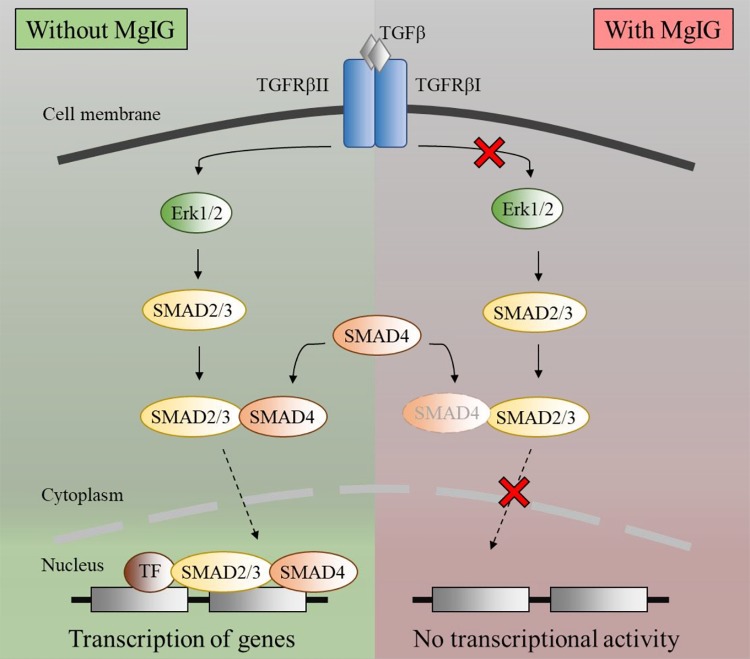
Schematic diagram of TGF-β pathway inhibited by MgIG. TGF-β binds and activates the ligand-activated TGFBR, thereby activating the ERK pathway. This process results in the phosphorylation of downstream SMAD2/3, which subsequently binds to SMAD4 before translocating into the nucleus. Together with multiple transcription factors, the SMAD2/3 and SMAD4 complex triggers the transcription of DNA. However, in the presence of MgIG, the phosphorylation of ERK as well as the binding of SMAD proteins are abolished, thereby leading to a decrease in transcriptional activity induced by the SMAD proteins.

Notably, the protein expression of αSMA did not reflect the decrease in its mRNA levels (**Figures 1A,B**) within the timeframe of our observation. This points toward the possibility that MgIG did not exhibit any direct effect on the protein levels, but only arrested SMAD-induced transcriptional activity which could innervate protein changes at a later timepoint. This data is in agreement with our observation on the cellular effects of MgIG in which there were no significant effects on proliferation, senescence and apoptosis of LX2 cells at 24 h treatment. These might suggest that the phenotypic changes mediated by MgIG may have a late onset. To further support this notion, we showed that MgIG could inhibit the upstream TGF-β signaling pathway by reducing the phosphorylation of ERK and binding of SMAD2/3 to SMAD4, thereby blocking the nuclear localization of the transcription factors (**Figure 7**). These initial events precede the suppression of fibrotic markers and the downstream cellular effects. Furthermore, inhibition of TGF-β signaling has been previously suggested as a potential mechanism to alleviate fibrogenesis (Dooley and ten Dijke, 2012; Tsuchida and Friedman, 2017). In other words, modulating TGF-β-activated HSCs using MgIG could offer a novel therapeutic approach to reverse hepatic fibrosis (Huang et al., 2017). Exactly how MgIG disrupts TGF-β signaling is still unknown, although we postulate that MgIG has an inhibitory effect on the major downstream pathways like the MAPK/ERK pathway. The downregulation of ERK phosphorylation could also explain the reduction in cellular proliferation (Ma et al., 2009). While we did not detect the activation of other molecular pathways involving Akt, p38, and JNK (**Figure 2A**), we reasoned that the use of low serum conditions to maintain the quiescent state of HSCs could be responsible in the low expression of their phosphorylated mediators. However, our study clearly delineated ERK phosphorylation as one of the downstream TGF-β signaling pathways in which MgIG targets.

Activated HSCs contributed largely to the inflammatory response during liver injury by producing various inflammatory molecules that interact with other cells in the liver (Weiskirchen and Tacke, 2014). On the other hand, they are also known to exhibit hepatoprotective effects by inhibiting chemokine and cytokine production (Fujita and Narumiya, 2016). Therefore, HSCs play an important role in regulating inflammatory response through their dual action in supporting liver inflammation or reducing parenchymal damage. As previously mentioned, the complete removal of HSCs by high concentrations of MgIG might not be feasible for anti-fibrotic treatment. Moreover, in terms of controlling inflammatory response in the liver, selecting the optimal concentration of MgIG is also crucial in substantiating the beneficial effect of MgIG in fibrosis. Previous studies have used lower concentrations of MgIG (below 5 mg/ml) to suppress inflammatory mediators such as reducing arachidonic acid metabolites production in macrophages (Xie et al., 2015) and inhibiting neutrophil cell infiltration (Wang et al., 2017). Furthermore, other studies have also shown that HSC senescence could limit liver fibrosis (Krizhanovsky et al., 2008; Nishizawa et al., 2016). Similarly, our results also showed that MgIG promotes senescence, induced limited cellular death and lowered caspase-3 activity in LX2 cells upon TGF-β activation (**Figures 5A,B**). The decrease in caspase 3 (**Figure 5A**) in LX2 cells was initially expected as a reduction in apoptosis of HSCs could potentially favor hepatic recovery. However, a higher concentration of MgIG induced cell death as shown by our PI staining (**Figure 5B**), suggesting that MgIG might have induced cell death of HSCs in alternative pathway. Recently, a new study has reported that MgIG instead induces ferroptosis in HSCs, which is a newly characterized mode of regulated cell death independent of caspase activity (Sui et al., 2018). Such pathway remains plausible and consistent in light of our observations reported herein. On the other hand, profound cellular death during acute liver injury reduces the ability of phagocytic cells to effectively remove dead tissues. As a result, apoptotic bodies start to accumulate and the production of pro-inflammatory factors increases, thereby worsening the fibrotic state of the liver (Guicciardi and Gores, 2010; Wang, 2014). Hence, a limited arresting or killing of activated HSCs while reverting some of them to their quiescent state is a favorable balance to aid in hepatic recovery. On the contrary, other studies have indicated that the apoptosis of HSCs could instead promote fibrosis reversion, given its prominent role in mediating hepatic fibrosis when in the activated form (Friedman, 2012; Troeger et al., 2012). While these findings proved that HSCs are important contributors of liver fibrosis, regression of the fibrotic state can be accounted for by either their senescence, reversion to quiescence or apoptosis. Hence, careful consideration should be placed on determining the optimal concentration of MgIG used to achieve HSC inactivation and reduce inflammatory conditions, while limiting extensive apoptosis of HSCs.

While MgIG is recognized for its hepatoprotective effect and anti-inflammatory effects, the possible side effects attributed to high concentrations of the drug should also be considered in assessing its suitability for use in the inflamed liver during fibrogenesis. We observed higher levels of caspase-3 activity in LO2 cells even at low concentrations of MgIG. However, we further argued that while these cells seemed to activate the apoptotic machinery, the cells did not exhibit higher cell death compared to activated LX2 cells when subjected to the same concentrations of MgIG. Therefore, this led us to hypothesize that (1) either LO2 cells were indeed more resistant to MgIG-induced cytotoxicity than LX2 cells or (2) that MgIG might indeed have cell specific hepatoprotective effects on LO2 cells, the latter being more plausible due to the number of mechanistic studies supporting its protective effects on hepatocytes (Zheng et al., 2015; Xu et al., 2016; Lu et al., 2017; Wu et al., 2018). In the context of liver fibrosis, as the injury persist, apoptotic hepatocytes may trigger the activation of HSCs and thus deteriorate the fibrotic condition (Puche et al., 2013). Hence, if indeed MgIG could protect hepatocytes from cellular death while eliminating activated HSCs, this could potentially translate to better fibrosis resolution and thus promote the use of MgIG as an anti-fibrotic drug. Future experiments assessing the cytotoxic profile of MgIG could be focused on other non-parenchymal cells of the liver such as Kupffer cells and sinusoidal endothelial cells to determine whether MgIG could be safely used, not only as an anti-inflammatory and hepatoprotective agent for the treatment of liver injury, but also as a potential anti-fibrotic drug to treat hepatic fibrogenesis.

## Conclusion

Our study proposed that low dose of MgIG could perturb the production of fibrotic markers and inhibit the proliferation of TGF-β-activated LX2 cells. We are first to report a plausible mechanism attributed to the inhibition of TGF-β-induced ERK pathway and subsequent reduction in nuclear localization of SMAD proteins. In addition, higher concentrations of MgIG have shown to inhibit proliferation, induce senescence and promote apoptosis in activated cells. These phenotypic changes have the potential to play an important role in ameliorating the microenvironment of the fibrotic liver. Since hepatic fibrosis is a dynamic process that involves various cellular and molecular changes, more studies are needed to further validate the effect of MgIG on the liver microenvironment. Nevertheless, our results provided insights into the mechanism of MgIG on HSCs and further promoted MgIG as a potential anti-fibrotic drug.

## Author Contributions

HH and BY conceived the hypothesis. JT and HH designed the research. JT and FP performed the studies on LX2. YT performed the studies on LO2. JT, FP, and YT analyzed the data. BY provided technical support. JT and HH wrote the manuscript.

## Conflict of Interest Statement

The authors declare that the research was conducted in the absence of any commercial or financial relationships that could be construed as a potential conflict of interest.
