# Supplementary material for: Magnesium Isoglycyrrhizinate Ameliorates Fibrosis and Disrupts TGF-β-Mediated SMAD Pathway in Activated Hepatic Stellate Cell Line LX2
Source: Front Pharmacol. 2018 Sep 25;9:1018. doi: 10.3389/fphar.2018.01018 (PMC6167412; doi:10.3389/fphar.2018.01018)
Supplement: Supplementary file 1 [file Data_Sheet_1.docx]

**Supplement Figure Legend****Figure S1 |** Detailed information of Magnesium Isoglycyrrhizinate Tetrahydrate

**Figure S2 |** TGF-β induced expression of fibrotic markers in hepatic stellate cells LX2 **(A)** Quantitative mRNA analyses revealed that TGF-β induced expression of fibrotic markers αSMA and collagen-1 in a dose-dependent manner after 24 hours treatment. DMEM with 10% FBS was used as a positive control. Data represents means ± s.e. of three biological replicates. *Two-tailed t-test with *p*-value < 0.05. **(B)** Western blot analyses detected increase in protein levels of αSMA and collagen-1 when cells were treated with TGF-β.

**Figure S3 |** MgIG did not reduce SMAD2/3 nuclear localization after 15 mins treatment duration. Immunofluorescence staining of TGF-β-activated LX2 cells after 15 mins treatment showed an observable increase in nuclear localization of SMAD2/3 as viewed under a confocal microscope at x20 and x60 magnification. 1 mg/ml MgIG did not reduce SMAD2/3 localization to the nucleus. SMAD2/3 antibody was tagged with Alexa488 (green), actin stained with CF568 phalloidin (red) and nucleus stained with Hoechst dye (blue). Scale bar = 50 µm.

**Figure S4 |** MgIG resulted in cell shrinkage of TGF-β-activated hepatic stellate cells LX2. Light microscopy detected morphological changes in TGF-β-activated LX2 cells treated with MgIG, particularly after 72 hours treatment, as compared to the non-treated (NegCtrl) and TGF-β treated cells at 24 hours. Scale bar = 20 µm.

**Figure S5 |** MgIG did not elicit any observable change in the morphology of fetal hepatocytes LO2. Light microscopy showed that LO2 cells retain their morphological phenotype despite the addition of 5.0 mg/ml MgIG up to 72 hours compared to the non-treated control (NegCtrl) at 24 hours treatment. Scale bar = 20 µm.

**Figure S6 |** Original images of Western Blot

Compound name: Magnesium Isoglycyrrhizinate Tetrahydrate

Chemical name: α-D-Glucopyranosiduronic acid, (3β, 18α, 20β)-2-0-carboxy-11-oxo-30-norolean-12-en-3-yl 2-O-β-D-glucopyranuronosyl-, magnesium salt, tetrahydrate

Chemical formula: C_42_H_60_MgO_16_ • 4H_2_O

Molecular weight: 917.28

Chemical structure:

**Supplement figure S1**


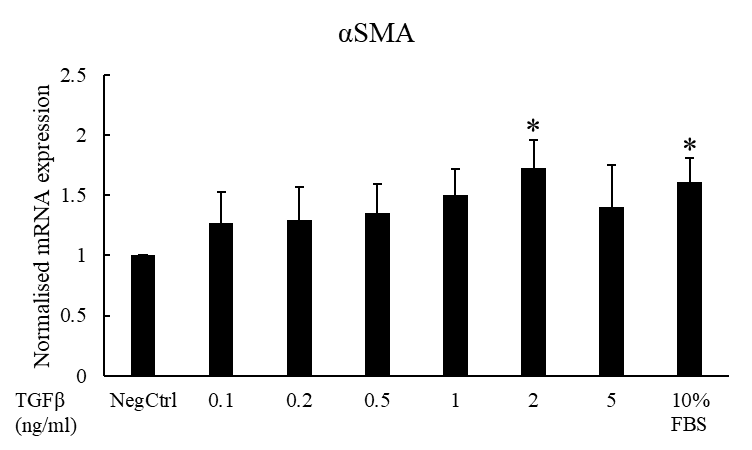


**A**

**B**


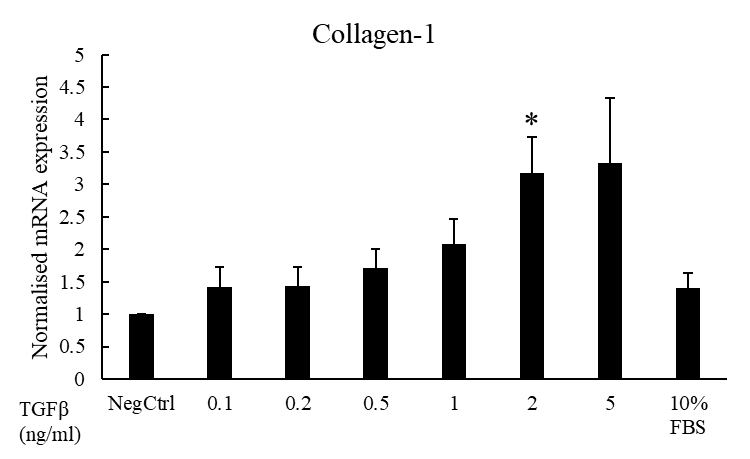

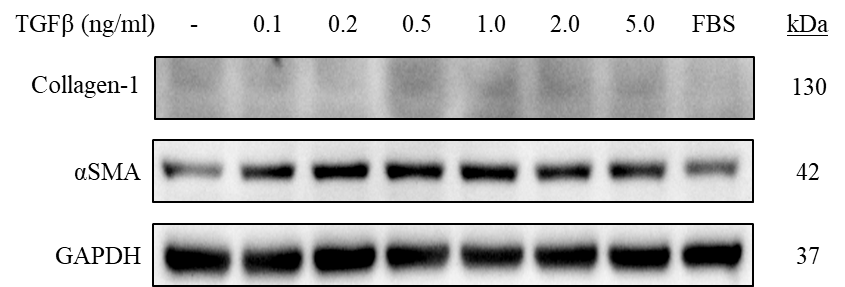


**Supplement figure S2**


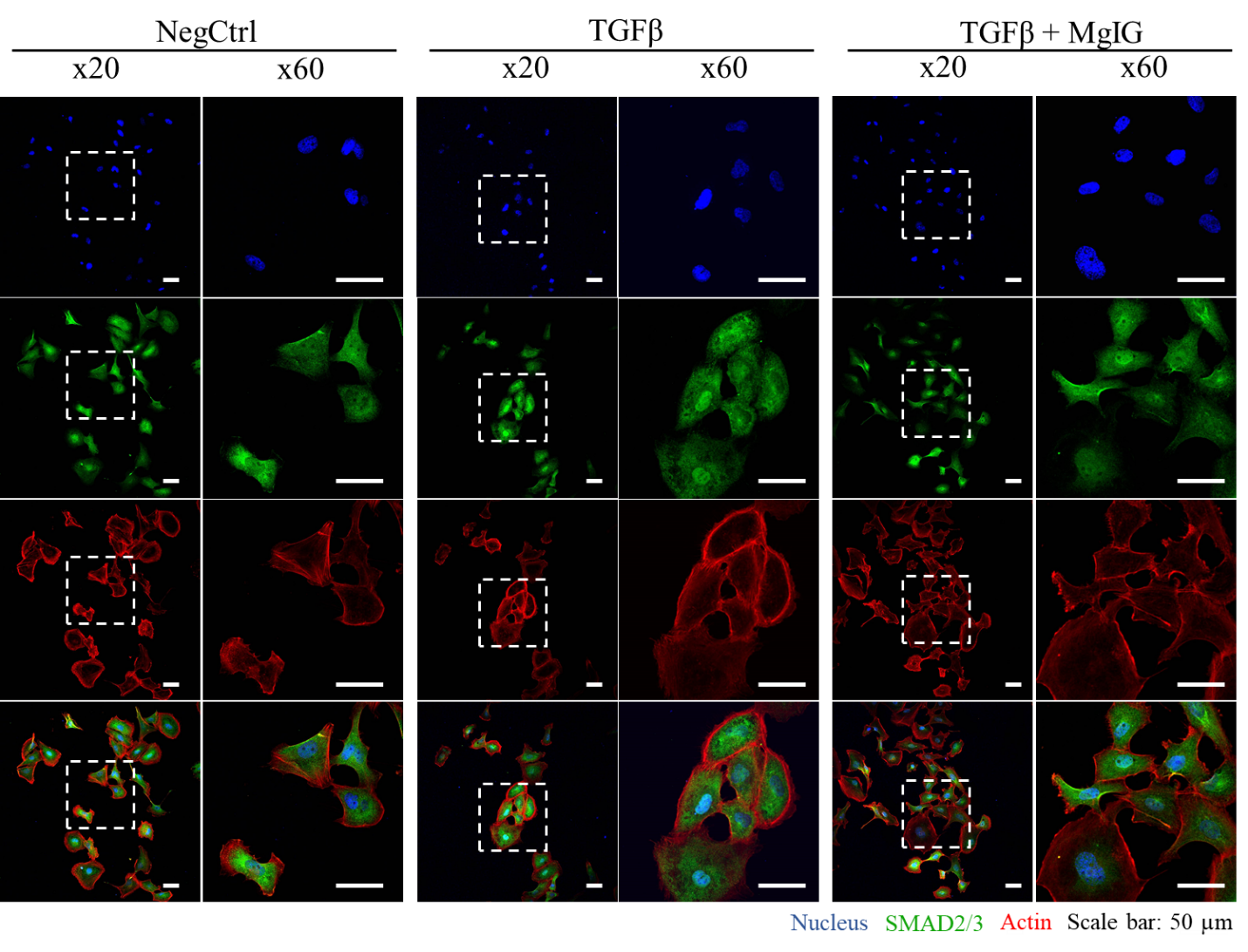
**Supplement figure S3**


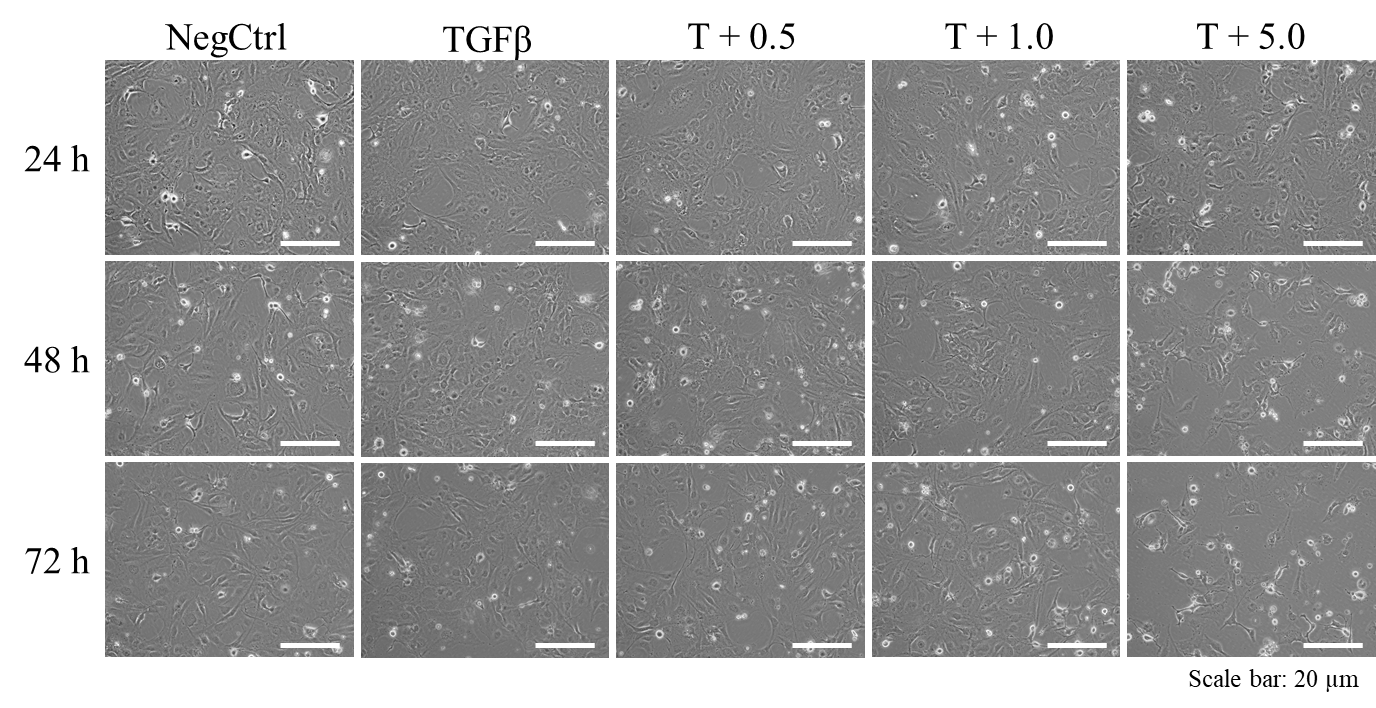
**Supplement figure S4**


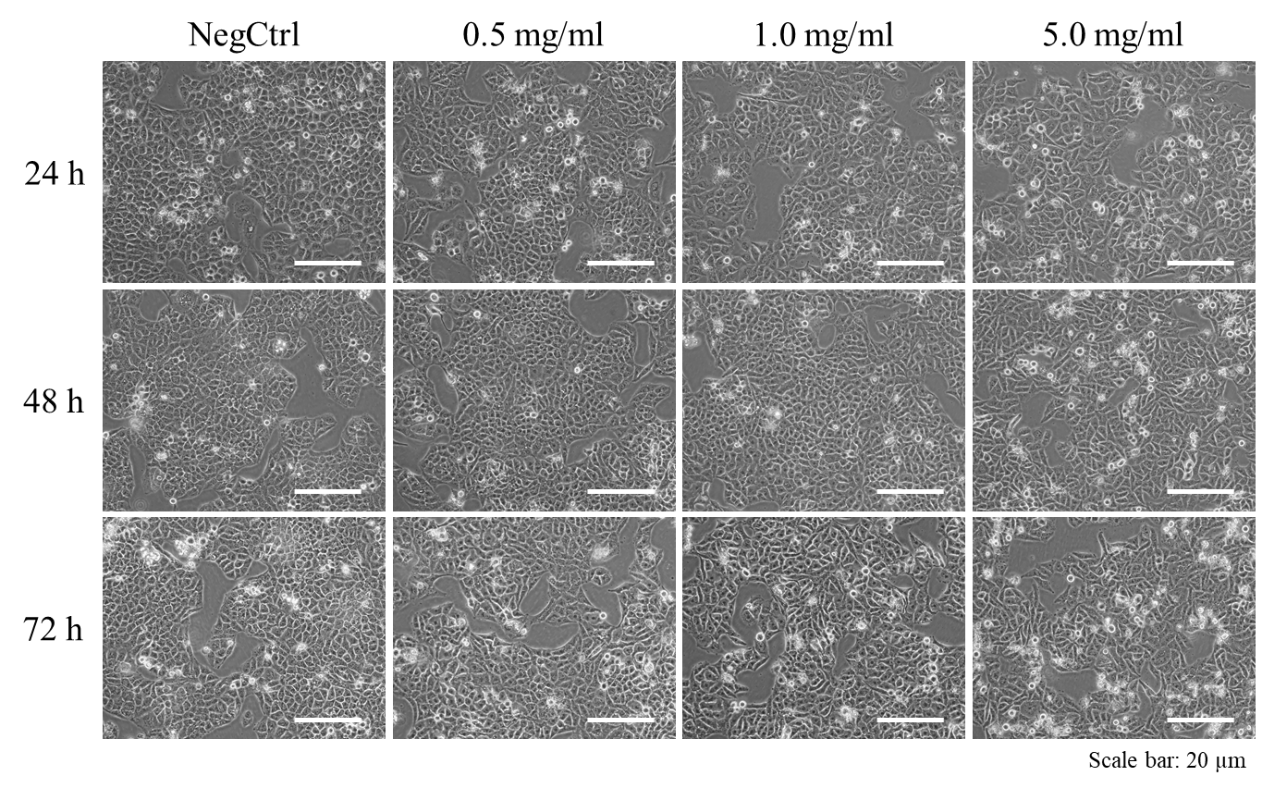


**Supplement figure S5**

**Figure S6 |** Original images of Western Blot

**
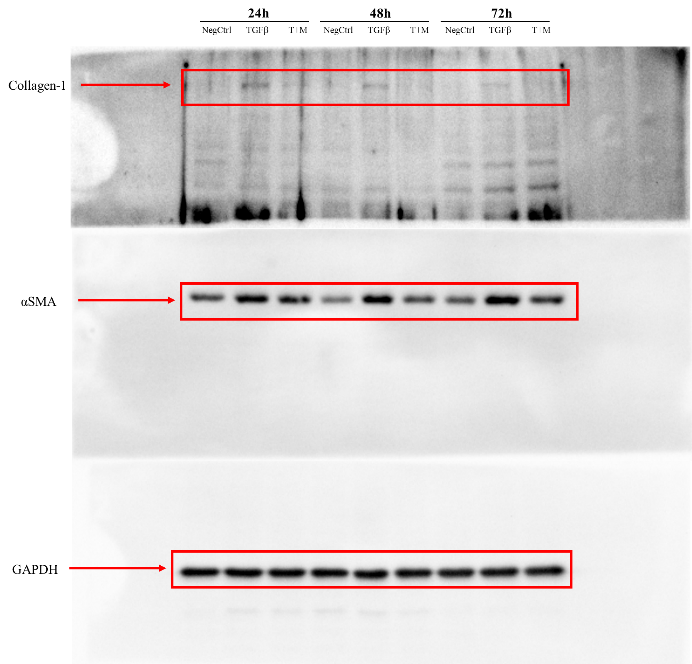
**

**Original images in Figure 1B**

**
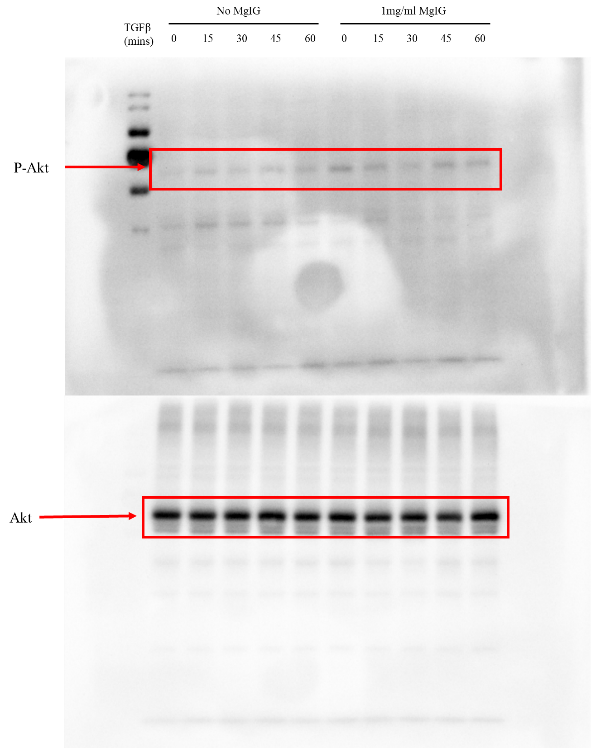

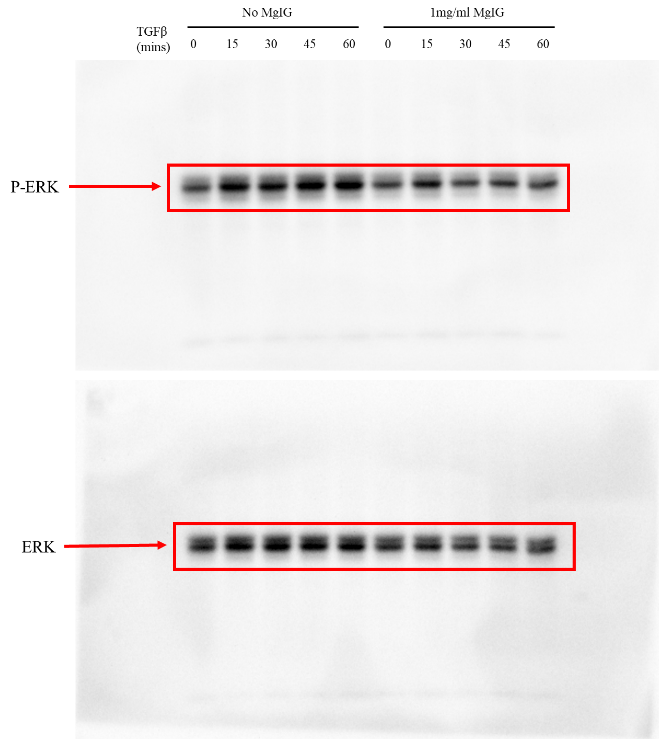
**

**
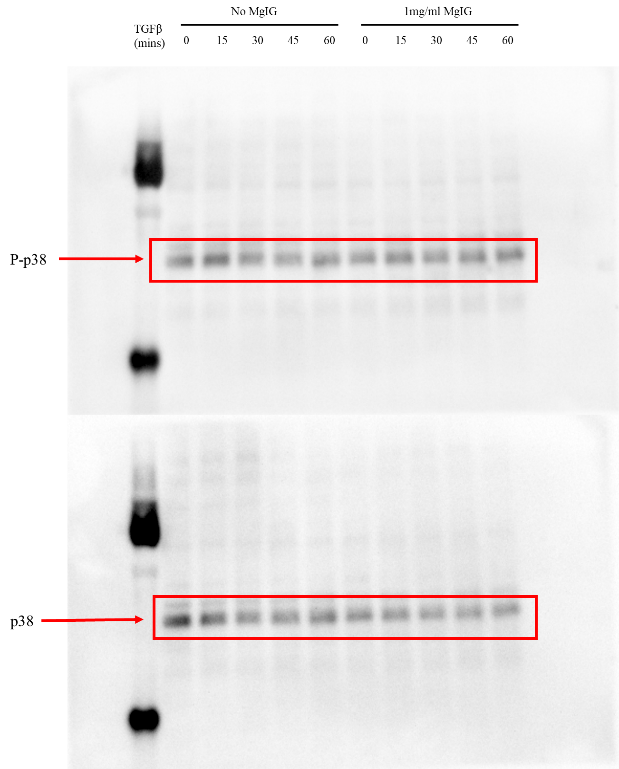

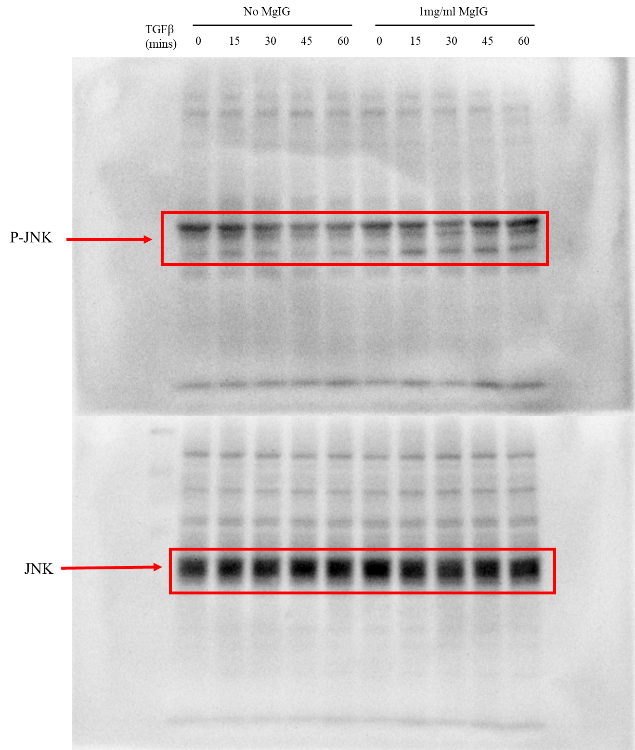
**

**Original images in Figure 2A**

**
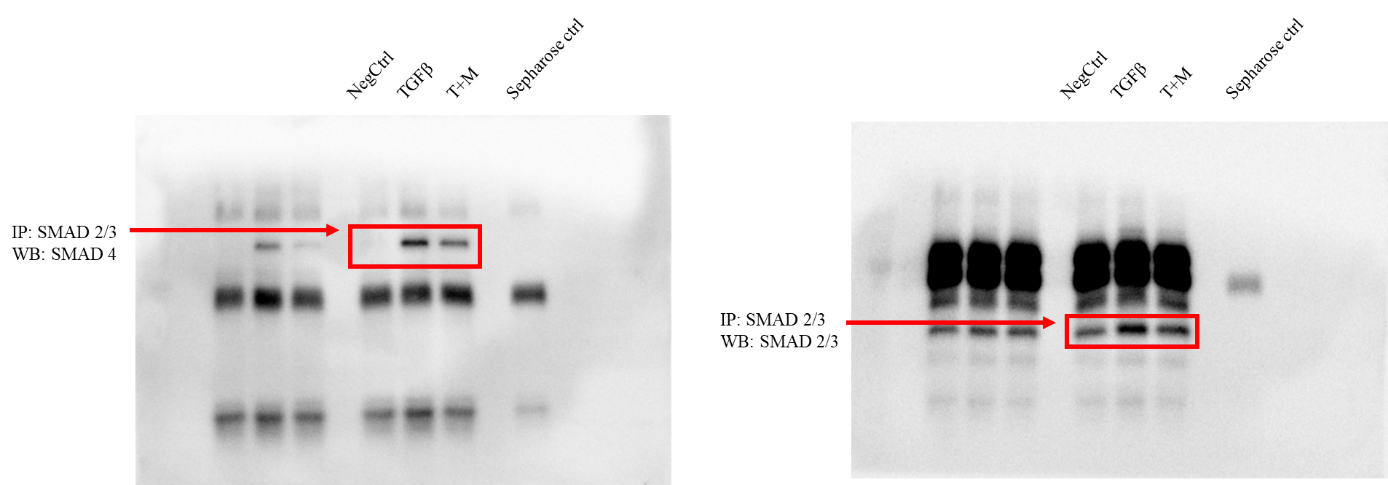
**

**Original images in Figure 2B**

**
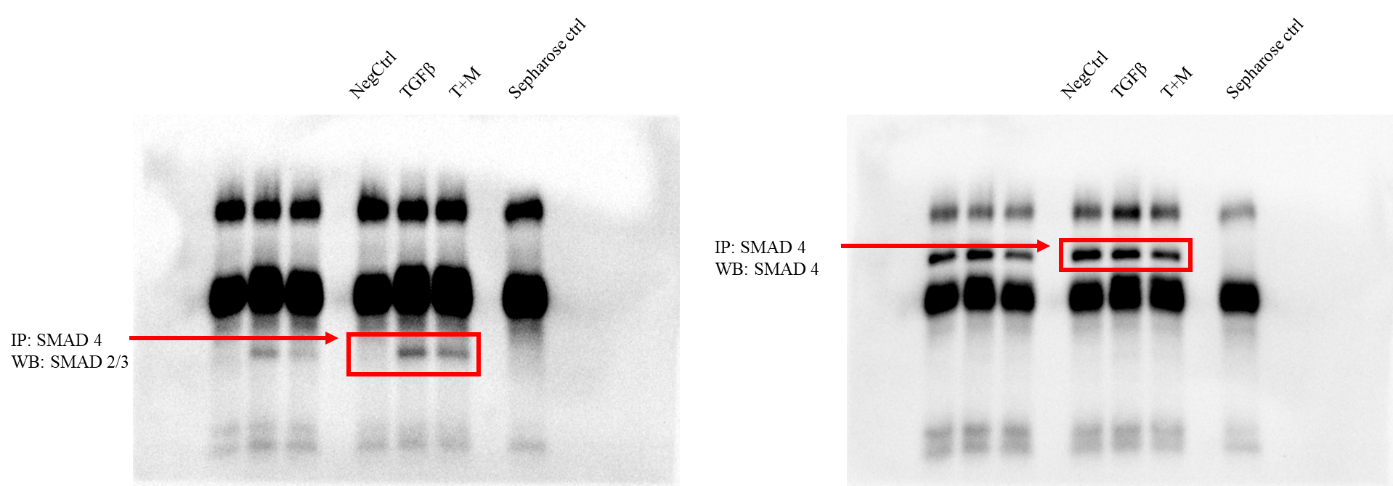
**

**Original images in Figure 2C**

**
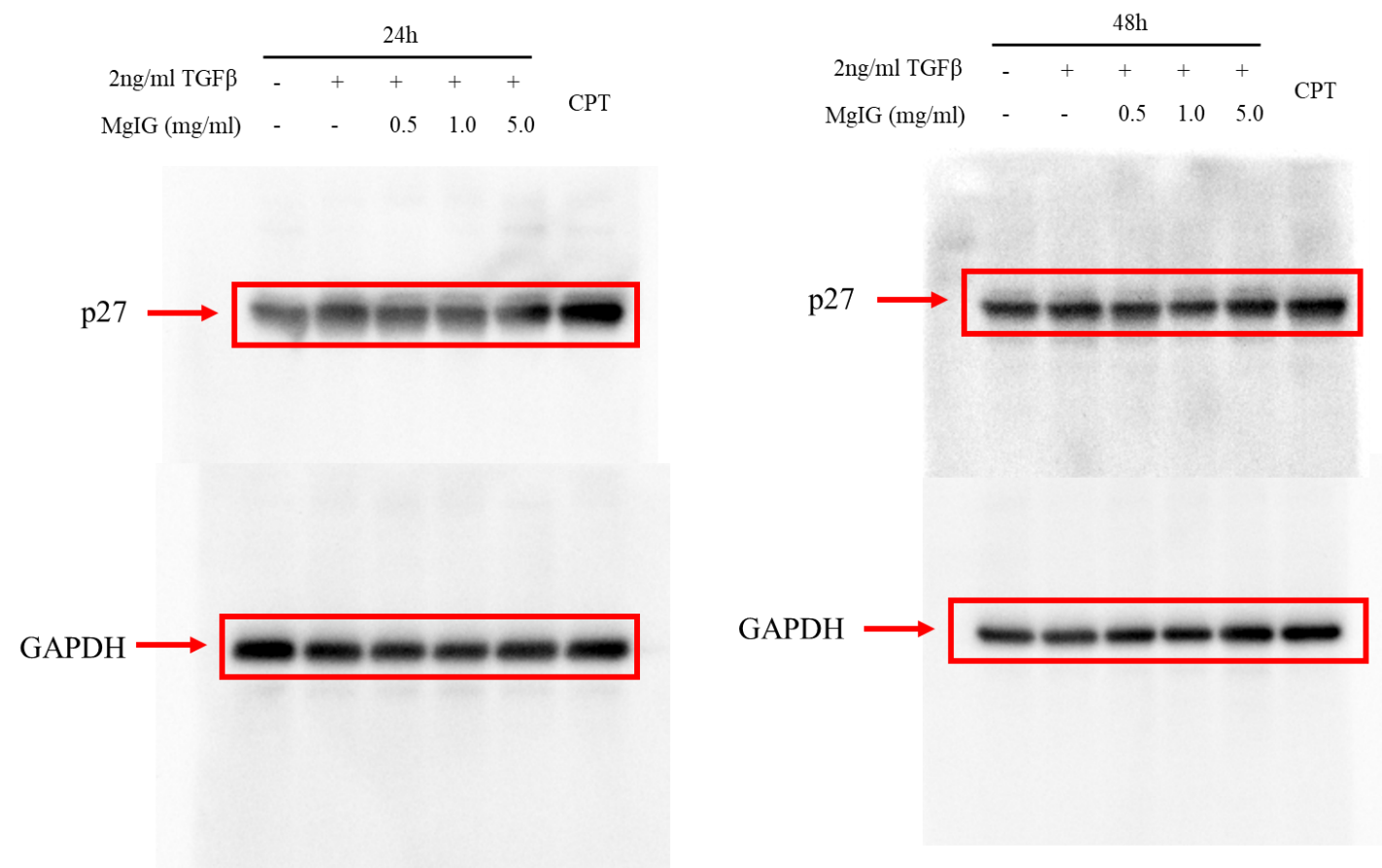
**

**Original images in Figure 4A**

**
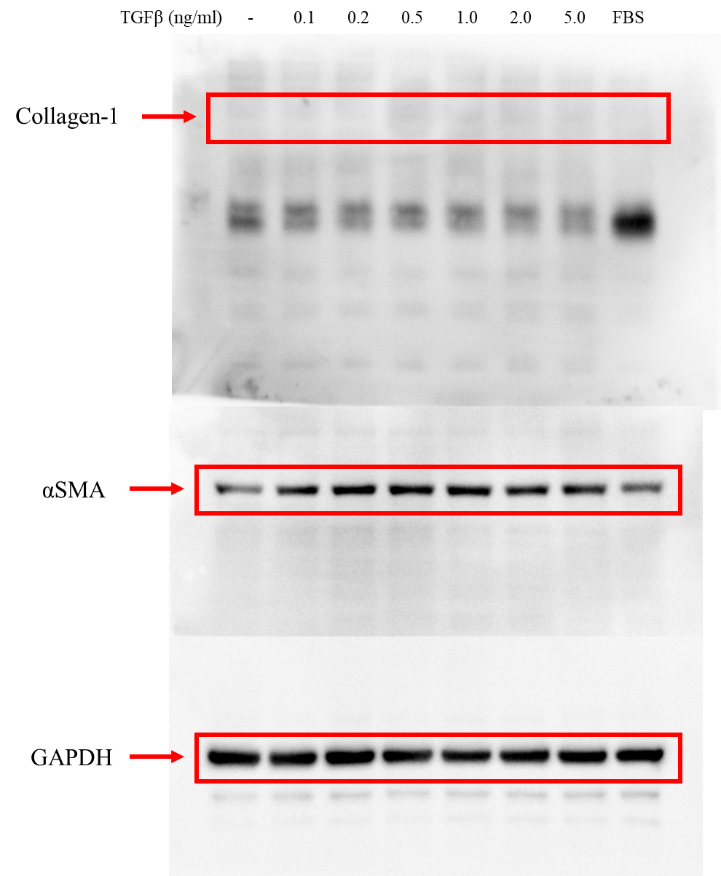
**

**Original images in Supplement figure S2B**
